# Longitudinal adherence trajectories measured by electronic dose monitoring and viral non-suppression in a South African HIV cohort study

**DOI:** 10.1186/s12879-026-13690-z

**Published:** 2026-06-11

**Authors:** Christopher M. Ferraris, Joseph G. Rosen, Campbell McDuling, Naomi Joy Fireman Schiavoni, Paul A. D’Avanzo, Lauren Jennings, Justin Knox, Robert H. Remien, Catherine Orrell

**Affiliations:** 1 New York State Psychiatric Institute, New York, NY USA; 2 School of Social Policy & Practice, University of Pennsylvania, Philadelphia, PA USA; 3 Division of General Internal Medicine, Rhode Island Hospital, Providence, RI USA; 4 Department of Medicine, The Warren Alpert Medical School, Brown University, Providence, RI USA; 5 Department of Epidemiology, School of Public Health, Brown University, Providence, RI USA; 6 Department of Statistics, University of Cape Town, Cape Town, South Africa; 7 Department of Psychology, Scripps College, Claremont, CA USA; 8 Rutgers School of Public Health, Rutgers University, New Brunswick, NJ USA; 9 Desmond Tutu HIV Centre, Institute of Infectious Diseases and Molecular Medicine, Department of Medicine, University of Cape Town, Cape Town, South Africa; 10 Department of Psychiatry, Irving Medical Center, Columbia University, New York, NY USA; 11 Department of Sociomedical Sciences, Mailman School of Public Health, Columbia University, New York, NY USA; 12 South African Medical Research Council, Cape Town, South Africa

**Keywords:** HIV; ART adherence; Electronic dose monitoring; Trajectory modeling

## Abstract

**Background:**

Sustained adherence to antiretroviral therapy (ART) is essential for maintaining viral suppression among people with HIV (PWH), yet adherence fluctuates over time and is imperfectly captured by routine viral load monitoring in many settings where measurements are often infrequent. We aimed to identify longitudinal ART adherence trajectories using electronic dose monitoring (EDM) and assess their association with viral non-suppression in a South African cohort.

**Methods:**

We analyzed data from a prospective cohort of PWH in Cape Town, South Africa on tenofovir-based ART. Daily adherence was measured using EDM over 12 months and summarized as monthly counts of dose events. Group-based trajectory modelling was applied to monthly adherence proportions to identify distinct adherence patterns. Associations between trajectory membership and time to viral non-suppression (HIV-1 RNA > 50 copies/mL) were assessed using Kaplan–Meier methods and log-rank tests.

**Results:**

Among 238 PWH, 5 adherence trajectories were identified: persistently high (49.6%), gradually declining (22.7%), delayed declining (9.6%), rapidly declining (8.0%), and consistently suboptimal adherence (10.1%). Rapidly declining and delayed declining trajectories were associated with earlier and higher incidence of viral non-suppression compared with persistently high and gradually declining trajectories (log-rank *p* < 0.001). Younger PWH were more likely to be in lower-adherence trajectories; baseline psychosocial characteristics did not differ across groups.

**Conclusions:**

ART adherence is dynamic and heterogeneous; early declines in electronically measured adherence are associated with increased viral non-suppression risk. Longitudinal adherence trajectories may offer clinically actionable signals before routine viral load testing, informing differentiated HIV care programmes.

## Introduction

The goal of antiretroviral therapy (ART) is to achieve and sustain viral suppression, which is predicated upon consistent daily adherence when a person with HIV (PWH) is prescribed daily oral ART. Adherence remains a significant challenge in many low- and middle-income countries (LMICs), including South Africa, where an estimated 7.8 million PWH reside [1]. Despite South Africa’s early national investment in universal ART access since 2004 [2], nearly 2 million people on treatment are not virally suppressed [1]. Routine viral load (VL) monitoring, the standard method for assessing ART adherence, is typically measured only annually after the first year of ART in South Africa, which may delay identification of ART adherence challenges or treatment failure [3]. These long intervals between VL assessments limit opportunities for timely ART adherence support and overlook potential virologic fluctuation that may occur between clinic visits. Additional tools that allow for improved monitoring of ART adherence could contribute to improved programme outcomes [4, 5].

Electronic dose monitoring (EDM) devices, such as Wisepill [6, 7], generate daily dosing data capable of identifying missed doses and short-term lapses in medication use [8, 9]. In South Africa, where durable ART adherence is essential to epidemic control, EDM devices represent a relatively low-cost option to monitor treatment engagement. Prior studies have demonstrated strong concordance between EDM indicators and biological adherence measures, including viral load suppression [10, 11] and drug concentrations from dried blood spots (DBS) [12], and have also been found to be highly acceptable among PWH in care [10, 13, 14]. Emerging research suggests that EDM devices also modestly improve adherence behaviors, [10] and PWH have also self-reported that using these devices helped them stay consistent with their ART use [14].

The strong concordance between EDM and biological measures of antiretroviral exposure supports its use as a viable proxy measure of ART adherence. Building on this evidence, several studies have applied trajectory modeling approaches to characterize longitudinal ART adherence patterns. EDM-based analyses among youth in the US [15] and South Africa [16] have identified multiple heterogeneous adherence trajectories. Studies using self-reported or prescription refill-based measures of ART use also show substantial variability in ART adherence over time [17–19], Taken together, these analyses demonstrate that trajectory modeling can detect early declines, sustained gaps, and other patterns indicative of HIV care discontinuity that single-time-point measures may miss.

In this study, we applied group-based trajectory modeling to 12 months of daily Wisepill-generated EDM data to identify distinct longitudinal adherence patterns among PWH prescribed tenofovir-based daily oral ART regimens in Cape Town, South Africa. We first characterized modeled adherence profiles over time and group-specific dosing patterns, then compared baseline sociodemographic and psychosocial characteristics across trajectory groups. We also assessed whether trajectory membership corresponded to differences in viral outcomes, including time to viral non-suppression. To our knowledge, this is one of the few studies to pair EDM trajectory modeling with viral suppression endpoints in an ART-experienced cohort. By integrating granular dosing data with longitudinal modeling and virologic outcomes, this study aims to deepen understanding of adherence dynamics and inform more responsive, data-driven strategies to achieve and sustain viral suppression in high-burden settings.

## Methods

Data for this study were drawn from a prospective research cohort that captured daily EDM data via Wisepill [7], and VL. Details of the parent study, which recruited 250 PWH in Cape Town from 2016 to 2020, have been published elsewhere [20]. A total of 250 PWH participants were sampled from four public-sector ART clinics in Cape Town, and all provided written informed consent in their preferred language (English or Xhosa). Participants attended scheduled monthly study visits over the 12-month follow-up period, during which viral load samples were collected and Wisepill adherence data were downloaded and devices serviced. These visits were part of the research protocol and did not necessarily correspond to routine ART refill or clinical care visits for stable patients in usual care. Eligibility requirements included: being 18 years of age or older, viral load suppression (< 50 RNA copies/ml) at enrollment, prescribed first-line TDF-based ART, and exhibiting elevated risk for viral non-suppression, defined as being in the first treatment year or having documented adherence challenges in the past year based on refill records, pill counts, chart reviews, electronic monitoring, or prior clinical evidence of viral non-suppression [20]. These clinical and adherence indicators were determined using available medical and study records and were used to identify individuals at elevated risk for adherence challenges despite viral suppression. This approach focused on PWH who were suppressed yet remained vulnerable to adherence challenges.

At baseline, comprehensive psychosocial and demographic data were collected, including validated measures of psychological distress (Kessler-10), mental health and substance use symptoms (SAMISS), HIV-related stigma, medication-specific social support, and HIV disclosure. These psychosocial measures were collected for analytic purposes and were not used to determine study eligibility or device assignment. These measures were part of the parent study’s assessment battery and were included because prior research has linked these psychosocial factors to ART adherence challenges and viral outcomes among people with HIV [21–23].

This study is reported in accordance with the Strengthening the Reporting of Observational Studies in Epidemiology (STROBE) guidelines.

### Measures

#### EDM

At baseline, participants were issued an EDM device containing a 30-day supply of their single-tablet ART regimen. The device transmits a time-stamped record of each daily opening (intake) or non-opening (heartbeat) through a cellular network, allowing continuous monitoring of dose-taking behavior. It is powered by a rechargeable battery with an approximate six-month lifespan. For this analysis, missed openings were defined as days on which no Wisepill opening was recorded, used as a proxy for a missed dose; multiple openings within a single day were treated as a single dose taken. These daily indicators were aggregated into monthly counts to characterize changes in adherence over time.

#### VL

At each monthly study visit, anticoagulated whole blood was collected for HIV-1 RNA quantification and processed by the National Health Laboratory Service using the Roche COBAS AmpliPrep/COBAS TaqMan HIV-1 assay.

#### Sociodemographics

At baseline, demographic information was obtained, including age, sex, education, and employment status. For analysis, age was summarized continuously, education was dichotomized as completion of secondary schooling or higher, and employment status was categorized as employed or unemployed.

#### Kessler 10

Psychological distress was assessed using the 10-item K10 [24], which measures nonspecific anxiety and depressive symptoms over the past 30 days. Items are rated on a 5-point scale and summed to a total score (10–50), with higher scores indicating greater distress. Established thresholds categorize distress as low (10–19), moderate (20–24), or high (≥ 25). The K10 has been previously validated in South Africa [25]. In this analysis, psychological distress was dichotomized to segment individuals with moderate-to-high distress from those exhibiting low distress.

#### SAMISS

The SAMISS is a 16-item screening tool for mental health and substance use among PWH [26], validated in South Africa [27]. It assesses mood, trauma, anxiety, depression, posttraumatic stress disorder (PTSD), and substance use. The five mental health and substance use components were aggregated and scored continuously.

#### HIV stigma

HIV-related stigma was measured using the 12-item short version of the HIV Stigma Scale [28]. Participants indicated agreement (1) or disagreement (0) with statements reflecting internalized, personalized, and disclosure-related stigma, and item scores were summed, with higher totals corresponding to higher levels of stigma. Stigma scores were then collapsed into a dichotomous measure, representing any versus no endorsement of stigma.

#### Life events questionnaire

Life events were measured at baseline using the 22-item Life Events Questionnaire (LEQ), an adapted version of the Stressful Life Events Screening Questionnaire [29].

#### Medication social support

Medication-related social support was assessed at baseline using an 8-item scale asking participants how often people in their lives assisted with HIV medication management. Items captured support such as monitoring symptoms, reminding medication intake, helping understand treatment information, encouraging communication with providers, and checking in about adherence. Responses ranged from 1 (“Never”) to 5 (“Very often”), with higher scores indicating greater medication-specific social support. Scores were then dichotomized to distinguish those reporting any medication social support from no medication social support.

#### HIV disclosure

HIV disclosure was measured by asking participants whether they had disclosed their HIV status to people in their social network (excluding healthcare providers). Items covered disclosure to a current partner, household members, family outside the home, friends, and coworkers, using categorical response options indicating the extent to which each group knew the participant’s HIV status. Items were aggregated, then collapsed into a dichotomous measure to capture individuals disclosing their HIV status to everyone in their respective social networks.

### Statistical analyses

Sociodemographic and psychosocial characteristics were summarized using proportions for categorical variables and medians with interquartile ranges for continuous measures. Group-based trajectory modeling was applied to monthly counts of days with recorded Wisepill openings, aggregated from daily EDM data over the first 12 months of observation. This approach allows identification of heterogeneous adherence patterns that may not be captured by population-average models. For descriptive presentation, these counts were also expressed as monthly adherence proportions. Latent adherence trajectories were derived solely from longitudinal EDM adherence data; baseline psychosocial and sociodemographic variables were not used in model estimation and were examined descriptively across trajectory groups after classification. Trajectory models were fit using finite mixture modeling, which identifies latent subgroups of individuals who share similar longitudinal adherence patterns [30]. A zero-inflated Poisson specification with a cubic polynomial for time was used to account for the non-negative count structure of the outcome and the high frequency of zero values. The zero-inflated component models the probability of excess zeros in the monthly count of openings; this parameter was specified as constant within trajectory groups. Exploratory examination of the monthly adherence counts indicated substantial zero inflation, and the zero-inflated Poisson formulation was, therefore, selected to accommodate excess zeros while allowing the mixture model to capture between-group variability in adherence patterns. Several functional forms for time (intercept-only, linear, quadratic, cubic, quartic, and quintic) were evaluated during model fitting. The cubic specification provided the best balance of model fit (based on Akaike and Bayesian Information Criteria), interpretability of trajectory shapes, and posterior probability diagnostics while avoiding overfitting observed with higher-order polynomials.

Competing models specifying different numbers of latent trajectory groups (2–7) were iteratively fit. Selection of the final model was guided by comparison of Akaike Information Criterion (AIC) values, Bayesian Information Criterion (BIC) values, entropy, and assessment of relative group sizes to avoid sparse trajectories [30, 31]. Once the optimal number of latent trajectories was identified, participants were assigned to a single trajectory based on the highest posterior probability of membership. Model adequacy was evaluated through visual inspection of each trajectory’s observed and model-predicted mean adherence values for interpretability and quality of longitudinal fit; range of confidence intervals for each estimated trajectory; average posterior probabilities of accurate trajectory classification; and odds of accurate trajectory classification [30, 31].

After trajectory classification, socio-demographic and psychosocial variables were compared across trajectory groups using Kruskal-Wallis tests for continuous variables and Fisher’s exact tests of association for categorical variables. Monthly VL measurements were used to construct time-to-event outcomes for HIV non-suppression, defined as *≥* 50 RNA copies/ml at first documented occurrence after baseline. Kaplan–Meier survival curves and log-rank tests were used to compare time to viral non-suppression across trajectory groups. Age-adjusted Cox proportional hazards models were then fit estimated to assess whether baseline age differences across trajectory groups, expressed as adjusted hazard ratios (aHR) with 95% confidence intervals (CI), influenced associations between trajectory membership and viral non-suppression. To assess the sensitivity of results to the virologic threshold implemented, a higher viral load cutpoint (*≥* 200 RNA copies/ml) was specified in subsequent models. All analyses were conducted using Stata/MP 18.0 [32].

## Results

Of the 250 participants enrolled, 238 PWH were included in the analytic sample. Twelve participants did not return for the first scheduled monthly follow-up visit (Visit 1) and therefore had no post-baseline adherence or viral load data; these individuals were excluded from trajectory and survival analyses.

Model fit indices supported selection of a five-group solution as the optimal balance of fit and parsimony, as improvements in AIC and BIC beyond five groups were accompanied by increasingly sparse class sizes (Tables 1 and 2). Classification diagnostics supported adequate model fit, with high agreement between maximum likelihood and posterior probability assignments, high average posterior probabilities (> 0.7), and strong odds of correct classification (> 4.00).

**Table 1:** Model fit statistics for group-based trajectory solutions (2–7 groups) of monthly ART adherence measured via electronic dose monitoring (*n* = 238)

No. of Latent Trajectories	Predicted Prevalence of Smallest Trajectory	Log Likelihood	Akaike Information Criterion (AIC)	Bayesian Information Criteria (BIC)	Entropy
2	28.3%	-18943.32	-18952.32	-18967.93	0.990
3	16.5%	-17354.29	-17368.29	-17392.57	0.983
4	6.8%	-16384.72	-16403.72	-16403.72	0.965
5	8.0%	-15861.18	-15885.18	-15926.79	0.965
6	4.2%	-15325.62	-15354.62	-15404.91	0.932
7	4.2%	-15184.30	-15218.30	-15277.26	0.930

Notes AIC and BIC values closer to 0 are favored fit in group-based trajectory modeling

**Table 2:** Performance and classification diagnostics for the five-trajectory group-based adherence model (*n* = 238)

Latent Trajectory	Proportion classified (MLE), 95% CI	Proportion classified (MPP)	Average Posterior Probability	Odds of Correct Classification
Consistently suboptimal (Group 1)	10.3% (6.2–13.9%)	10.1%	0.990	99.65
Rapidly declining (Group 2)	8.0% (4.5–11.5%)	8.0%	0.999	1394.84
Delayed declining (Group 3)	9.7% (5.9–13.5%)	9.7%	0.999	2470.25
Gradually declining (Group 4)	23.1% (17.5–28.7%)	22.7%	0.989	93.91
Persistently high (Group 5)	49.1% (42.5–55.7%)	49.6%	0.985	67.69

Notes MLE = Maximum Likelihood Estimation, MPP = Maximum Posterior Probability. Criteria for model adequacy include: high agreement between proportion classified to each trajectory group by MLE and MPP, reasonably narrow confidence intervals relative to the size of the trajectory group, average posterior probability of correct trajectory group classification > 0.7, and odds of correct trajectory group classification > 4.00

### Adherence trajectory patterns and viral outcomes

Group-based trajectory modeling identified five distinct adherence patterns over the 12-month period, described in Table 3. Nearly half of participants (Group 5, 49.6% prevalence) maintained persistently high adherence (> 90% monthly openings), while 22.7% (Group 4) exhibited high but gradually declining adherence. Three smaller subgroups demonstrated lower adherence patterns: consistently suboptimal adherence (Group 1, 10.1% prevalence), rapidly declining adherence (Group 2, 8.0% prevalence), and delayed decline after an initially high adherence at baseline (Group 3, 9.6% prevalence). Median monthly adherence levels ranged from 13.8% to 93.1% across groups, reflecting substantial heterogeneity in longitudinal dosing behavior. The proportion exhibiting HIV non-suppression at the end of the 12-month observation period was highest in the rapidly declining group (16.7%), followed by the delayed decline (13.6%), whereas individuals with gradually declining or persistently high adherence exhibited the lowest propensity for non-suppression at endline (2.0% and 3.6%, respectively).

**Table 3:** Characteristics of group-based adherence trajectories among adults with HIV receiving ART in Cape Town, South Africa (*n* = 238)

Group	*N* (%)	Trajectory Pattern	Median Monthly EDM (IQR)	Pattern Summary	% with viral non-suppression (VL *≥* 50) over 12-month follow-up
Group 1	24 (10.1%)	Consistently suboptimal	36.7% (18.2–73.3%)	Continuously low (< 50%) over time	4.6%
Group 2	19 (8.0%)	Rapidly declining	13.8% (3.5–69.2%)	Accelerated declining adherence	16.7%
Group 3	23 (9.6%)	Delayed declining	50.0% (27.5–71.0%)	Initially high, followed by a rapid decline	13.6%
Group 4	54 (22.7%)	Gradually declining	72.4% (58.6–83.9%)	Initially high and very slow decline	2.0%
Group 5	118 (49.6%)	Persistently high	93.1% (85.7–97.1%)	Consistently high (> 90%) over time	3.6%

Notes IQR= interquartile range; ART= antiretroviral therapy; VL= viral load; EDM= electronic dose monitoring

### Baseline sociodemographic and psychosocial characteristics

Across the 238 participants, sociodemographic characteristics varied significantly by adherence trajectory group (displayed in Table 4). Age differed significantly across groups (*p* < 0.001), ranging from 27 years in the rapidly declining group (Group 2) to 36.5 years among those with persistently high adherence (Group 5). Women comprised the majority across trajectory groups (65.2%–87.5%), with no significant differences by sex (*p* = 0.433). Educational attainment and employment were also similar across groups, with roughly one-third of participants having completed secondary education or higher (*p* = 0.167) and 12.5%–36.4% reporting current employment (*p* = 0.171).

**Table 4:** Baseline Sociodemographic and Psychosocial Characteristics of Adults with HIV Receiving ART in Cape Town, South Africa, by Adherence Trajectory Group (*n* = 238)

Variable	Consistently suboptimal (Group 1) (*n* = 24)	Rapidly declining (Group 2) (*n* = 19)	Delayed declining (Group 3) (*n* = 23)	Gradually declining (Group 4)(*n* = 54)	Persistently high (Group 5) (*n* = 118)	*p* -value
Age (med, IQR)	28.5 (25.5–39)	27 (23–32)	32 (26–37)	33 (26–41)	36.5 (31–45)	**< 0**.**001**
Female	21 (87.5%)	16 (84.2%)	15 (65.2%)	41 (75.9%)	92 (78.0%)	0.433
Education ≥ Secondary	5 (20.8%)	8 (44.4%)	4 (17.4%)	21 (38.9%)	43 (36.4%)	0.167
Employed	3 (12.5%)	4 (21.1%)	7 (30.4%)	17 (31.5%)	43 (36.4%)	0.171
K10	6 (25.0%)	3 (15.8%)	5 (21.7%)	11 (20.4%)	27 (22.9%)	0.969
HIV Stigma	17 (70.8%)	14 (73.7%)	19 (82.6%)	45 (83.3%)	95 (80.5%)	0.675
SAMISS Score	5 (0–10)	6 (0–10)	5 (1–11)	4.5 (0–10)	2 (0–7)	0.415
Life Events Score	2 (1-3.5)	2 (0–3)	1 (0–4)	2 (1–3)	2 (1–3)	0.720
Medication Social Support	21 (87.5%)	18 (94.7%)	20 (87.0%)	48 (88.9%)	94 (80.7%)	0.401
HIV Disclosure Comfort	14 (58.3%)	7 (36.8%)	13 (59.1%)	23 (42.6%)	52 (45.6%)	0.440

Notes: IQR= Interquartile range; ART= antiretroviral therapy; EDM= electronic dose monitoring; SAMISS = Substance Abuse and Mental Illness Symptoms Screener; VL= viral load. P-values were calculated using Kruskal–Wallis tests for continuous variables and Fisher’s exact tests for categorical variables

Psychosocial characteristics did not differ significantly across trajectory groups. Indicators of psychological distress, HIV-related stigma, mental health issues, substance use, stressful life events, medication-specific social support, and comfort with HIV disclosure were broadly comparable. Although proportions and median values varied modestly, none of these measures exhibited statistically significant group-level differences (all *p* > 0.40).

### Modeled longitudinal adherence profiles

**Fig. 1:**
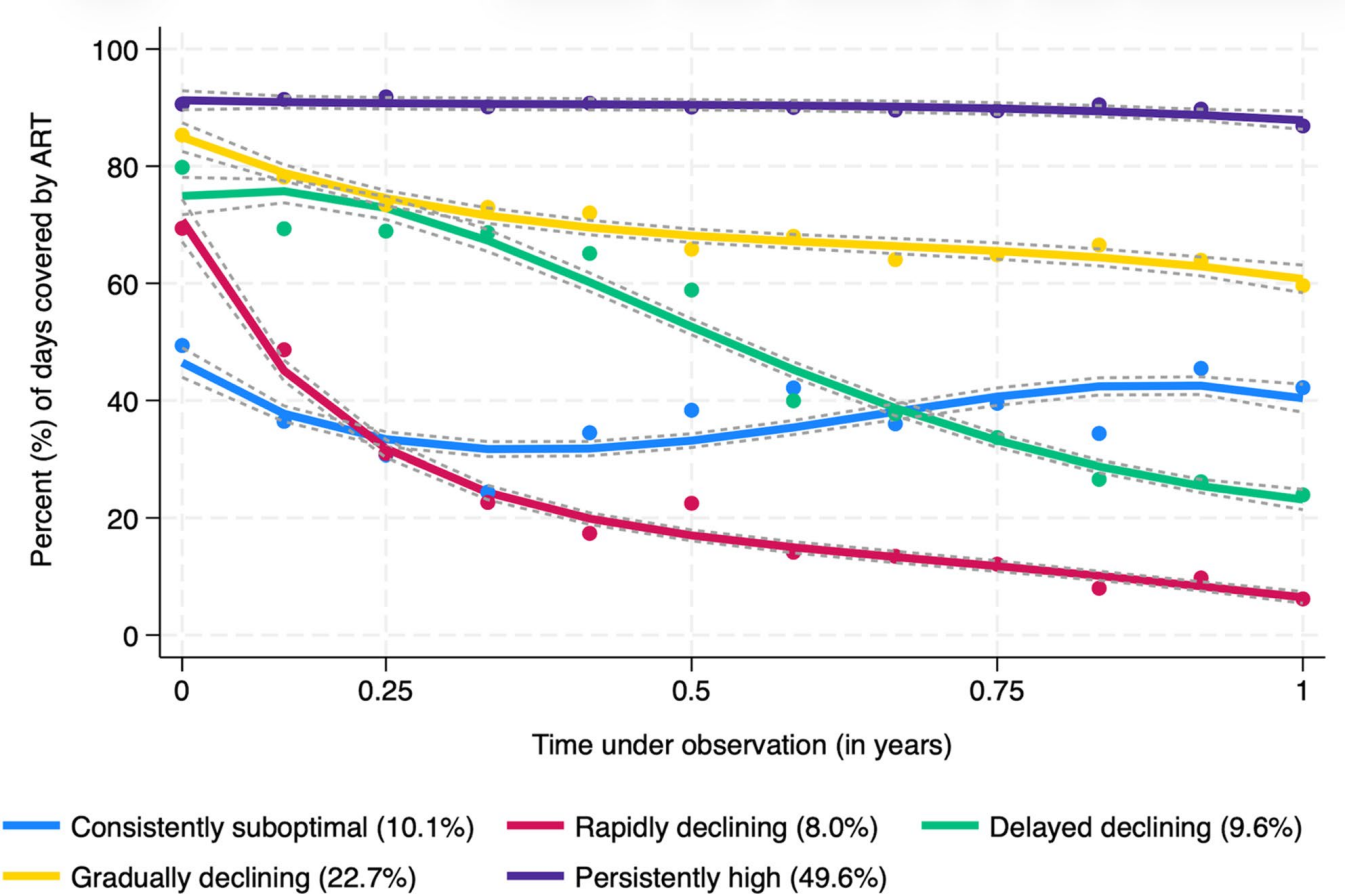
Electronic dose monitoring trajectories identified via group-based trajectory modeling among people with HIV receiving ART in Cape Town, South Africa (*n* = 238). Trajectories were estimated using monthly adherence proportions derived from electronic dose monitoring (EDM) device openings over 12 months. Models were based solely on EDM data; viral load measures were not included in trajectory estimation. The figure displays modeled longitudinal adherence patterns derived from monthly EDM adherence proportions over time

Figure 1 illustrates five distinct longitudinal adherence trajectories derived from group-based modeling. Nearly half of participants were assigned to a persistently high-adherence trajectory, while a smaller subset demonstrated high adherence with a gradual decline over time. The remaining groups showed lower and more unstable dosing patterns, including consistently low adherence, rapid early decline, and delayed decline following initially strong adherence.

### Time to viral non-suppression by adherence trajectory

**Fig. 2:**
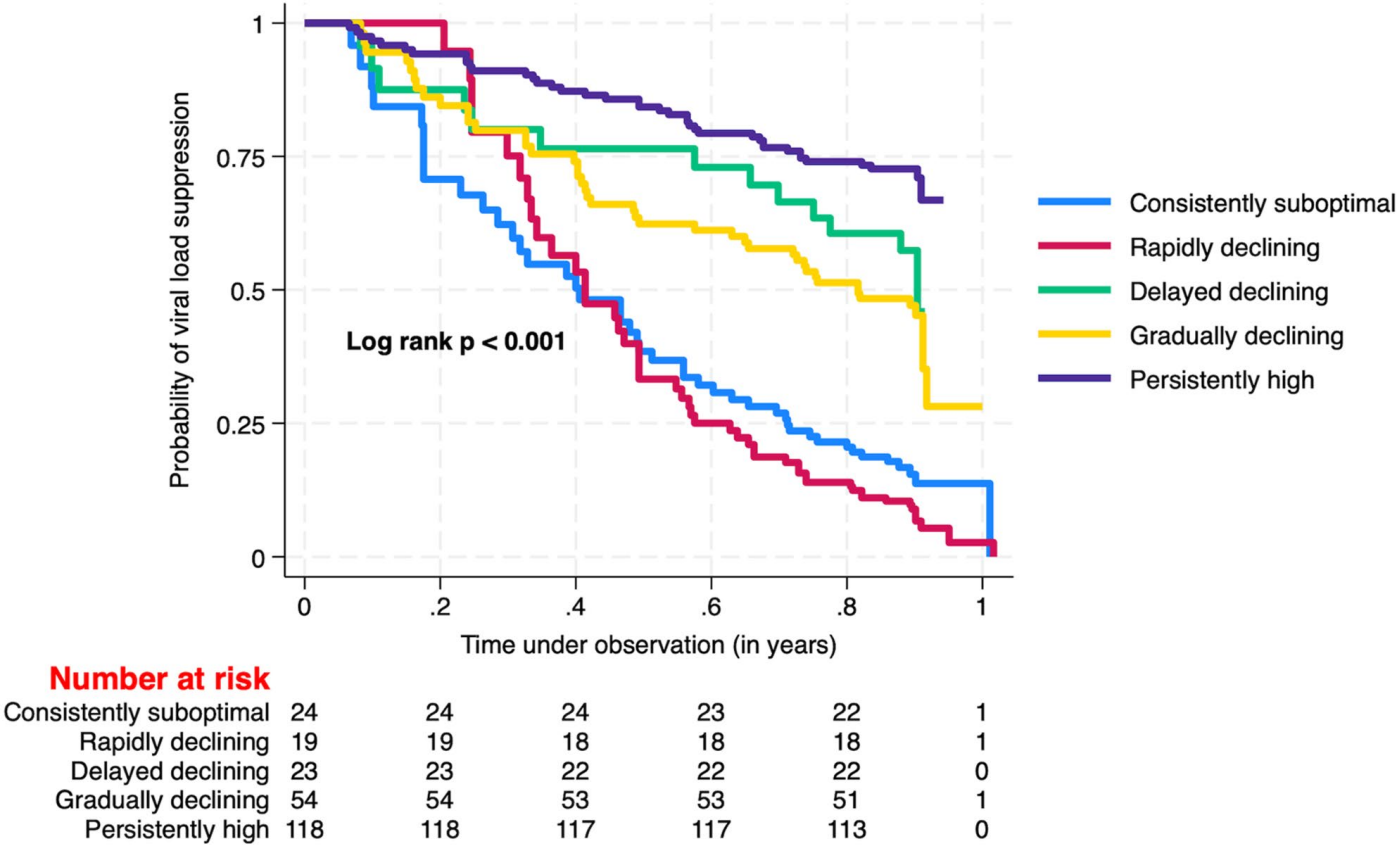
Kaplan–Meier estimates of time to viral non-suppression by adherence trajectory group among people with HIV receiving ART in Cape Town, South Africa (*n* = 238). Trajectory group membership was defined using EDM-derived adherence patterns over 12 months. Kaplan–Meier curves display time to viral non-suppression (HIV-1 RNA >50 copies/mL) by trajectory group; viral load data were not used to define trajectories. Declines in numbers at risk at the far right of the figure reflect the administrative end of study follow-up rather than participant attrition or incomplete data collection

Figure 2 displays Kaplan–Meier survival curves estimating time to viral non-suppression across adherence trajectory groups. Participants in the persistently high-adherence and gradually declining groups maintained the highest probability of sustained viral suppression over follow-up, with only small proportions experiencing non-suppression. In contrast, the rapidly declining and delayed declining adherence trajectories exhibited earlier and steeper reductions in suppression probabilities, corresponding to a higher incidence of viral non-suppression events. The consistently suboptimal adherence trajectory demonstrated an intermediate pattern, with modest declines over time. Overall, the survival curves indicate that lower or declining adherence trajectories were associated with accelerated times to viral non-suppression (log-rank *p* < 0.001), reinforcing the clinical relevance of early trajectory divergence for anticipating virologic risk. Age-adjusted Cox proportional hazards models yielded similar results, with trajectory membership remaining associated with viral non-suppression risk and age itself not significantly associated with non-suppression (Table 5).

**Table 5:** Age-adjusted hazard ratios (aHR) and 95% confidence intervals (CI) of viral non-suppression, by discrete suppression cutpoints

Covariates	*≥* 50 copies/ml		*≥* 200 copies/ml	
	aHR (95% CI)	*p*-value	aHR (95% CI)	*p*-value
Latent trajectory				
Consistently suboptimal (Group 1)	**5.94 (1.28–27.57)**	**0.023**	**23.73 (4.94-114.11)**	**< 0.001**
Rapidly declining (Group 2)	**6.45 (1.37–30.42)**	**0.019**	**28.20 (5.66-140.53)**	**< 0.001**
Delayed declining (Group 3)	1.00	*Ref*.	2.52 (0.23–28.03)	0.453
Gradually declining (Group 4)	2.78 (0.63–12.34)	0.178	**6.71 (1.34–33.58)**	**0.020**
Persistently high (Group 5)	1.18 (0.27–5.26)	0.821	1.00	*Ref*.
Age, in years	1.02 (0.99–1.05)	0.132	0.99 (0.95–1.04)	0.807

Notes: The reference category in each Cox proportional hazards model was identified based on the lowest hazard of viral non-suppression

## Discussion

This study used group-based trajectory modeling of EDM ART adherence data among a cohort of PWH in Cape Town and identified 5 distinct ART adherence trajectories, which ranged from persistently high adherence to rapidly declining and consistently suboptimal use, thus highlighting the dynamic nature of ART adherence over time. Different trajectories were associated with differential risk of viral non-suppression: PWH with early and sustained declines in ART adherence experienced accelerated times to loss of viral suppression. While most sociodemographic and psychosocial indicators did not significantly differentiate adherence groups, age was a notable exception, with younger individuals having lower adherence trajectories, underscoring the vulnerability of younger adults to suboptimal treatment outcomes, even after achieving viral suppression initially.

The five identified trajectories reflect both stability and vulnerability within the cohort. Nearly half of participants sustained consistently high adherence, suggesting a group well-suited to differentiated, lower-intensity care. In contrast, others followed more fragile paths. Gradually and delayed declining adherence patterns may reflect emerging or progressive disengagement over time, while rapidly declining and consistently suboptimal adherence trajectories point to individuals at elevated risk of HIV care disengagement. By integrating VL outcomes into trajectory modeling, the analysis provides clinically actionable insight. However, the adherence trajectories identified in this study were derived retrospectively using 12 months of follow-up data and should be interpreted as descriptive rather than real-time classification tools. These adherence patterns are not uniform in onset or severity, underscoring the importance of real-time monitoring to detect early changes in behavior that precede viral non-suppression. Methodologically, these analyses contribute to the literature by integrating daily EDM data into longitudinal assessments of daily ART adherence, providing a more granular depiction of private pill-taking behaviors than is possible with periodic, interval-based measures (e.g., self-report, pharmacy refills, or VL).

These findings reinforce growing evidence that adherence is not a static behavior but unfolds along heterogeneous paths. Studies across HIV care contexts, including ART cohorts in sub-Saharan Africa as well as global pre-exposure prophylaxis (PrEP) trials and demonstration studies, have consistently identified subgroups with high, declining, or persistently low adherence [15, 16, 33], often with important implications for virologic and prevention outcomes. Although many prior studies have relied on VL or pharmacy refills data [16, 34], the use of EDM in our analysis adds temporal precision, capturing nuanced adherence patterns preceding suboptimal clinical outcomes and signal opportunities for timely intervention. Trajectory modeling should be viewed as complementary to simpler adherence metrics, such as average or recent adherence, which remain clinically useful and more readily implemented. However, trajectory-based approaches may better capture sustained patterns or changes in adherence over time that are not reflected in summary measures alone.

Across diverse HIV populations, roughly half of individuals tend to maintain consistently high adherence, while the remainder follow more fragile or declining patterns over time. Our trajectories reflect other data from the Western Cape Provincial Health Data Centre, where 59% had optimal engagement with the ART programme over a year; and 38% over 5 years [35]. In a large adolescent cohort in the Eastern Cape, nearly half sustained strong adherence, whereas the rest demonstrated increasing, decreasing, or persistently low trajectories [16]. Similar distributions across other settings and regions have been observed in other ART cohorts [18, 19], underscoring that heterogeneous adherence patterns are common across diverse treatment settings. Our findings align with this structure, capturing both gradual and abrupt declines in adherence. The absence of “improving” adherence trajectories in our cohort may reflect that all participants were virally suppressed at enrollment and already well-engaged in care (despite signals of adherence challenges), making downward patterns more likely to emerge. This mirrors prior longitudinal ART studies, which similarly found that declining adherence trajectories tend to dominate among adults with initially optimal adherence [15, 19].

Finally, age emerged as a key factor in adherence patterning, with younger adults overrepresented in lower adherence trajectories. This is consistent with evidence from both ART [34, 36, 37] and PrEP [38, 39] studies indicating that younger PWH face elevated challenges with sustained adherence. Importantly, prior trajectory analyses have also found that younger individuals are more likely to cluster into lower-adherence or declining adherence groups, including studies in adolescents, young adults, and adult ART cohorts [16, 18, 19]. As differentiated care models expand, trajectory-based monitoring may help identify younger patients and others trending toward disengagement earlier, allowing for more proactive and tailored adherence support. Notably, baseline psychosocial characteristics did not differ across trajectory groups. This may reflect unmeasured or time-varying factors, as well as the limitations of baseline measures in capturing dynamic adherence processes.

For clinicians, these findings offer actionable insight into how adherence monitoring can be used to inform patient management. While age remains an important marker, especially among younger adults who showed higher likelihood of suboptimal adherence, this study highlights that risk is also embedded in behavioral patterns over time. These differences in time to viral non-suppression across trajectory groups support the clinical relevance and differentiation of the identified adherence patterns. Findings were consistent across both > 50 and > 200 copies/mL thresholds, supporting robustness to outcome definition. Trajectories marked by early or sharp declines in adherence may warrant closer monitoring or additional support, even in the absence of elevated viral loads. Incorporating longitudinal adherence data into clinical decision-making could help identify patients in need of intervention before virologic failure occurs. In settings with limited resources, trajectory-informed approaches may help prioritize adherence counseling, digital outreach, or more frequent follow-up for those whose patterns suggest future risk of HIV care discontinuity, while safely streamlining care for those on consistently strong adherence paths.

### Limitations and strengths

This study has several limitations. First, generalizability is limited by the study context, as participants were virally suppressed at baseline and drawn from an urban South African cohort, albeit one experiencing documented adherence challenges. Second, EDM devices such as Wisepill have generally been found acceptable and useful for supporting medication adherence in South Africa and other high-burden settings [40, 41]. However, experiences with digital adherence technologies are mixed, with some individuals reporting concerns related to device visibility, stigma, or feeling monitored [42, 43]. In this study, use of Wisepill was part of participation in the parent research cohort, and individuals unwilling to use electronic monitoring were not enrolled; therefore, the sample may reflect participants more comfortable with such technologies. Notably, adherence still declined in the trajectory groups despite this potential selection toward greater device acceptability. Third, while EDM provides high-resolution data on pill-taking behavior, it does not confirm ingestion and may over- or under-estimate adherence in cases where participants removed multiple pills at once or opened the device without taking medication. Additionally, trajectory membership was assigned based on maximum posterior probability following model estimation; although posterior probabilities were high across groups, downstream analyses treated class membership deterministically and therefore did not explicitly account for residual classification uncertainty. Fourth, although we integrated viral load outcomes, the study was not powered to detect differences in clinical events beyond viral non-suppression, such as CD4 cell count changes or drug resistance emergence. Finally, psychosocial measures were collected only at baseline and may not have captured dynamic changes that could influence adherence behavior over time. Future work could further examine within-person dosing gaps and prolonged stretches without Wisepill openings to better characterize temporal patterns of non-adherence captured by EDM. Such approaches may also support the development of early or dynamic classification strategies using partial adherence data. Additionally, clinic-level retention outcomes such as loss to follow-up were not available in this analysis, limiting our ability to assess whether lower-adherence trajectories corresponded to disengagement from care.

Despite these limitations, this study offers several important strengths. The use of daily EDM data over an extended 12-month period allowed for fine-grained, longitudinal assessment of adherence behavior, capturing patterns that traditional self-report or refill-based metrics might miss. By applying group-based trajectory modeling, we were able to empirically identify distinct adherence subgroups rather than rely on arbitrary thresholds. The integration of objectively measured viral load outcomes strengthens the clinical relevance of these patterns, linking behavioral trajectories with virologic risk. Although some trajectory groups were relatively small, the overall sample was sufficient to demonstrate heterogeneity in longitudinal adherence patterns and their association with viral non-suppression. Importantly, this analysis represents one of the few applications of electronic adherence trajectory modeling within an ART cohort in sub-Saharan Africa, offering a novel perspective on adherence dynamics in a high-burden setting.

## Conclusion

This study identified distinct adherence trajectories among South African adults on ART using high-resolution electronic monitoring, with each trajectory associated with different risks of viral non-suppression. While all participants were virally suppressed at baseline, inclusion required evidence of adherence vulnerability, allowing for meaningful variation in behavioral patterns over time. By linking longitudinal adherence profiles with virologic outcomes, this analysis contributes to a growing body of evidence that adherence is dynamic and clinically consequential. It further extends the literature by applying group-based trajectory modeling to EDM data in a South African treatment cohort, offering a more granular understanding of adherence behavior than is possible with traditional metrics. These findings support the integration of trajectory-informed approaches into differentiated care models, enabling earlier identification of patients at risk of viral non-suppression and more timely, tailored adherence support.

## Data Availability

The datasets used and/or analyzed during the current study are not publicly available due to ethical and privacy restrictions but are available from the corresponding author on reasonable request and subject to approval by the study investigators and relevant ethics committees. Deidentified individual participant data and a data dictionary will be provided for approved research purposes. The study protocol and statistical analysis plan are available from the corresponding author upon request.

## Abbreviations

ART: Antiretroviral therapy
BIC: Bayesian Information Criterion
DBS: Dried blood spots
EDM: Electronic dose monitoring
HIV: Human immunodeficiency virus
IQR: Interquartile range
K10: Kessler Psychological Distress Scale (10-item)
LMICs: Low- and middle-income countries
MLE: Maximum likelihood estimation
MPP: Maximum posterior probability
PWH: People with HIV
SAMISS: Substance Abuse and Mental Illness Symptom Screener
STROBE: Strengthening the Reporting of Observational Studies in Epidemiology
TDF: Tenofovir disoproxil fumarate
TFV-DP: Tenofovir diphosphate
VL: Viral load
